# Genome size variation and karyotype diversity in eight taxa of *Sorbus* sensu stricto (Rosaceae) from China

**DOI:** 10.3897/CompCytogen.v15i2.58278

**Published:** 2021-05-20

**Authors:** Jiabao Li, Kailin Zhu, Qin Wang, Xin Chen

**Affiliations:** 1 Co-Innovation Center for Sustainable Forestry in Southern China, College of Biology and the Environment, Nanjing Forestry University, Nanjing, 210037, Jiangsu, China Nanjing Forestry University Nanjing China

**Keywords:** DNA content, flow cytometry, polyploid, *Sorbus* evolution

## Abstract

Eight taxa of *Sorbus* Linnaeus, 1753 sensu stricto (Rosaceae) from China have been studied karyologically through chromosome counting, chromosomal measurement and karyotype symmetry. Genome size was also estimated by flow cytometry. Six taxa, *S. amabilis* Cheng ex T.T.Yu et K.C.Kuan, 1963, S. hupehensis var. paucijuga (D.K. Zang et P.C. Huang, 1992) L.T. Lu, 2000, *S. koehneana* C.K. Schneider, 1906, *S. pohuashanensis* (Hance, 1875) Hedlund, 1901, *S. scalaris* Koehne, 1913 and *S. wilsoniana* C.K. Schneider, 1906 are diploids with 2n = 34, whereas two taxa, *S. filipes* Handel-Mazzetti,1933 and *S. ovalis* McAllister, 2005 are tetraploid with 2n = 68. In general, the chromosome size is mainly small, and karyotypes are symmetrical with predominance of metacentric chromosomes. Genome size variation of diploids and tetraploids is 1.401 pg –1.676 pg and 2.674 pg –2.684 pg, respectively. Chromosome numbers of *S. amabilis* and S. hupehensis var. paucijuga, and karyotype and genome size of eight taxa studied are reported for the first time. This study emphasised the reliability of flow cytometry in genome size determination to infer ploidy levels in Chinese native *Sorbus* species.

## Introduction

*Sorbus* Linnaeus, 1753 (sensu stricto, except as noted hereafter) (Maleae, Rosaceae) is distributed mainly in northern temperate regions with its greatest diversity in the mountains of south-western China and adjacent areas of Upper Burma and the Eastern Himalaya. It comprises about 90 species all over the world, with more than 60 species occurring in China (Phipps et al. 1990; Lu and Spongberg 2003; McAllister 2005). Species of *Sorbus* are valuable ornamental plants due to their pinnately compound leaves, attractive white or red flowers and colourful crimson, scarlet, orange, pink, yellow or pure white fruits. *Sorbus* is one of the most challenging groups in taxonomy and systematic for the widespread interspecific hybridisation and genome multiplication (polyploidy) (McAllister 2005; Robertson et al. 2010; Li et al. 2017). Polyploidy is a very common phenomenon in the genus. Tetraploids account for more than half of the species richness and are distributed mainly in the mountains of south-western China, especially the Qinghai-Tibet Plateau (McAllister 2005). Thus, data of chromosome number and ploidy levels in Chinese native *Sorbus* species are valuable in the taxonomy of the genus and in understanding the species’ relationships and origins.

Features of chromosomes play an important role in plant taxonomy to elucidate the origin, speciation and phylogenetic relationships of plants (Stebbins 1971; Peruzzi and Altınordu 2014; Sassone et al. 2017; Winterfeld et al. 2020). Chromosome counts were proven to be most valuable in the taxonomy of *Sorbus* long before the era of molecular phylogenetics because they are helpful in understanding the species’ relationships and origins (Liljefors 1934, 1953, 1955; Sennikov and Kurtto 2017). The chromosome base number in *Sorbus* is x = 17 and it is common to all members of Maleae. Chromosome counts have been reported for 43 Chinese native *Sorbus* species. Only two ploidies occur in the genus, i.e. diploid (2n = 34) and tetraploid (2n = 68), although four ploidies have been reported in *Sorbus* sensu lato (McAllister 2005; Bailey et al. 2008; Pellicer et al. 2012). Most species occur at one ploidy level, and two Chinese native species, *S. koehneana* C.K. Schneider, 1906 and *S. vilmorinii* C.K. Schneider, 1906, have been reported to have diploids and tetraploids (McAllister 2005).

Genome size estimation (plant genome C-value) by flow cytometry (FCM) (Greilhuber et al. 2005) is a rapid cytogenetic method that has contributed to our understanding of the evolutionary relationships amongst *Sorbus* species (Hajrudinović et al. 2015a, b). FCM profiles revealed the presence of two ploidy levels (cytotypes) in the genus, 2n = 2x (*S. cibagouensis* H. Peng et Z. J. Yin, 2017: 1.480 ± 0.039 pg, *S. hypoglauca* (Cardot, 1918) Handel- Mazzetti, 1933: 1.513 ± 0.041 pg) and 2n = 4x (*S. vilmorinii*: 2.675 ± 0.065 pg) (Xi et al. 2020), consistent with the results of chromosome counts (Pellicer et al. 2012).

The present study aims to (1) determine the chromosome number, karyotype, idiogram and other chromosome morphology and genome size of eight taxa in *Sorbus*; and (2) evaluate the reliability of flow cytometry in genome size determination to infer ploidy levels in Chinese *Sorbus* species.

## Materials and methods

### Plant material

**Figure 1.**
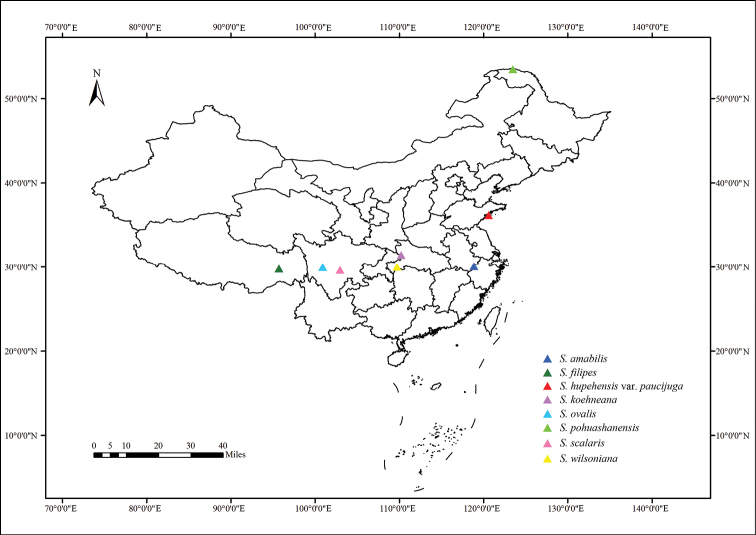
Collection sites of the eight *Sorbus* taxa studied.

Eight taxa from two subgenera in *Sorbus*, *S. filipes* Handel-Mazzetti, 1933, S. hupehensis var. paucijuga (D.K. Zang et P.C. Huang, 1992) L.T. Lu, 2000, *S. koehneana*, *S. ovalis* McAllister, 2005 from subgenus Albocarmesinae McAllister, 2005 and *S. amabilis* Cheng ex T.T.Yu et K.C.Kuan, 1963, *S. pohuashanensis* (Hance, 1875) Hedlund, 1901, *S. scalaris* Koehne, 1913, *S. wilsoniana* C.K. Schneider, 1906 from subgenus Sorbus, were collected in China (Figure 1) between 2015 and 2016. Three individuals for each taxon were selected for chromosome numbers counting, karyotype analysis and genome size estimation. Voucher specimens are deposited at the Herbarium of Nanjing Forestry University (**NF**).

### Chromosome preparations and karyotype analysis

Mature fruits of each plant were harvested separately, then plump seeds were extracted from fruits and washed with tap water. Seeds were stored in sand for 40–120 days at 0–4 °C until germination. Root tip meristems were pre-treated with a mixed solution of 0.1% colchicine and 0.002 mol/l 8-hydroxyquinoline (1:1) at 0–4 °C for 2 h and then fixed in absolute ethanol: glacial acetic acid (2:1) mixture for 24 h at 0–4 °C. The root tips were hydrolysed in 1 mol/l HCl at 60 °C for 10min and then rinsed with tap water for 2–3 min. The fixed roots were stained in Carbol fuchsin for 3–4 h, ground and placed on glass slides for observation. Five metaphase cells per individual were examined. Photos were taken under an optical microscopic Nikon Eclipse Ci-S. A mean haploid idiogram was drawn using KaryoType 2.0 (http://mnh.scu.edu.cn/soft/blog/karyotype/, Altinordu et al. 2016), based on the length of chromosome.

For the numerical characterisation of the karyotypes, the following parameters were calculated: long arm length (LA) and short arm length (SA) of each chromosome, ratio of the longest/shortest chromosomes(L/S), total haploid (monoploid) length of chromosome set (THL), arm ratio of each chromosome (AR) [LA/SA], centromeric index of each chromosome (CI) [SA/ (LA + SA) × 100] and chromosome length of each chromosome (CL) [LA + SA]. Karyotype asymmetry has been determined using the coefficient of variation of centromeric index (CV_CI_) [(S_CI_ / X_CI_) × 100, where S_CI_: standard deviation; X_CI_: mean centromeric index] (Paszko 2006), coefficient of variation of chromosome length (CV_CL_) [(S_CL_ / X_CL_) × 100, where S_CL_: standard deviation; X_CL_: mean chromosome length] (Paszko 2006) and Stebbins’ classification (Stebbins 1971). The karyotype formula was determined by chromosome morphology based on centromere position according to Levan et al. (1964): median point (M, AR = 1.00), median region (m, AR = 1.01–1.70), submedian (sm, AR = 1.71–3.00), subterminal (st, AR = 3.01–7.00) and terminal region (t, AR > 7.00). Satellite chromosomes were abbreviated as ‘sat’ (Levan et al. 1964). In terms of length, chromosomes were classified according to Lima de Faria (1980) as very small (≤ 1 µm), small (> 1 µm and = ≤ 4 µm), intermediate (> 4 µm and = < 12 µm) and large (> 12 µm and ≤ 60 µm).

### Genome size estimation

Fully expanded leaf tissue from each sample collected in the field was dried in silica gel. Approximately 1 cm^2^ of the sample was chopped along with the internal standard [Oryza sativa subsp. japonica S. Kato, 1930 ‘Nipponbare’, 2C = 0.91 pg, (Uozu et al. 1997)] using a sharp razor blade in a Petri dish containing 1 ml of ‘woody plant buffer’ (WPB, Loureiro et al. 2007), following the one-step procedure proposed by Doležel et al. (2007). The nuclear suspension was then filtered through a nylon mesh (400 μm) to remove debris and stained with 50 μl PI. After incubation for 10 min on ice, the relative nuclear DNA content was estimated by recording at least 3000 particles using a BD Influx flow cytometer ﬁtted with a blue laser (488 nm, 200 mW) and analysing three replicates of each individual. The resulting histograms were analysed with the BD FACS software 1.0.0.650. The 2C-value was calculated using the linear relationship between fluorescence signals from stained nuclei of the unknown sample and the internal standard. 1Cx was calculated dividing the 2C-value by the ploidy.

### Statistical analysis

Data were analysed with SPSS Statistics 22.0 (IBM, USA). Correlations between chromosome counts and 1Cx, 2C-value were assessed using the Pearson correlation coefﬁcient.

## Results and discussion

**Figure 2.**
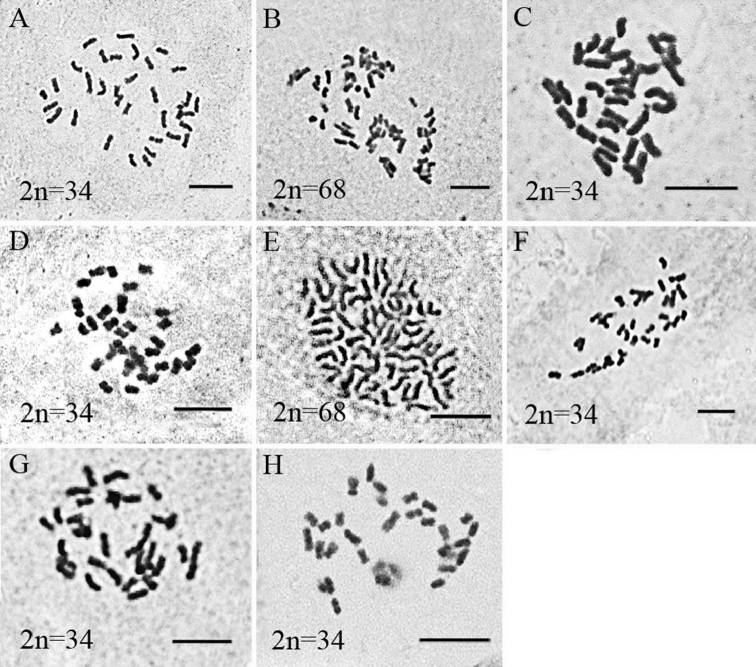
Somatic metaphases of eight *Sorbus* taxa **A***S. amabilis***B***S. filipes***C**S. hupehensis var. paucijuga**D***S. koehneana***E***S. ovalis***F***S. pohuashanensis***G***S. scalaris***H***S. wilsoniana*. Scale bar: 5 μm.

The chromosome numbers of eight Chinese taxa of *Sorbus* in two subgenera have been determined (Table 1). All taxa have the same base chromosome number (x = 17). Four taxa, *S. amabilis* (Fig. 2A), *S. pohuashanensis* (Fig. 2F), *S. scalaris* (Fig. 2G) and *S. wilsoniana* (Fig. 2H) belonging to subg. Sorbus, are all diploids with 2n = 2x = 34. Amongst the taxa studied in subgen. Albocarmesinae, two taxa, S. hupehensis var. paucijuga (Fig. 2C) and *S. koehneana* (Fig. 2D), are diploids, while two other taxa, *S. filipes* (Fig. 2B) and *S. ovalis* (Fig. 2E), are tetraploids with 2n = 4x = 68. Chromosome numbers of two taxa, *S. amabilis* and S. hupehensis var. paucijuga, are reported for the first time. The chromosome numbers of six other taxa are consistent with the results of previous studies (Lu and Spongberg 2003; McAllister 2005).

**Table 1.** Collecting information of materials and cytogenetics data of studied *Sorbus* taxa.

Subgenera	Taxon	2n	L/S	THL (µm)	VCL (µm)	MAR	X_CI_ (%)	CV_CI_	CV_CL_	Haploid karyotype formula	Stebbins’ classiﬁcation	2C (pg, mean ± s.d.)	1Cx (pg)
Subgenus Albocarmesinae	*S. filipes*	68	2.49	26.63	0.98–2.12	1.63	38.68	12.98	21.11	10m (1sat) + 7sm	2B	2.684 ± 0.042	0.671
	S. hupehensis var. paucijuga	34	2.13	31.50	1.15–2.41	1.68	37.61	9.75	14.03	10m (1sat) + 7sm	2B	1.407 ± 0.007	0.704
	*S. koehneana*	34	2.27	22.18	0.89–1.79	1.33	43.36	9.47	19.20	15m + 2sm	1B	1.571 ± 0.029	0.785
	*S. ovalis*	68	2.29	31.84	1.19–2.52	1.19	45.85	4.86	18.12	17m	1B	2.674 ± 0.015	0.669
Subgenus Sorbus	*S. amabilis*	34	2.49	37.38	1.54–3.73	1.71	38.87	21.54	24.70	9m +7sm (1sat) +1^st^	2B	1.401 ± 0.026	0.700
	*S. pohuashanensis*	34	2.08	50.06	2.05–4.08	1.46	41.35	13.23	16.66	13m (1sat) + 4sm	2B	1.664 ± 0.052	0.832
	*S. scalaris*	34	2.10	29.03	1.14–2.39	1.58	39.47	14.44	19.39	13m (1sat) + 4sm	2B	1.676 ± 0.044	0.838
	*S. wilsoniana*	34	1.95	20.68	0.89–1.72	1.25	44.84	9.57	18.37	16m + 1sm	2A	1.556 ± 0.089	0.778

L/S: Ratio of the longest/shortest chromosomes; THL: Total haploid (monoploid) length of chromosome set; VCL: Variation in chromosome length; MAR: Mean arm ratio; X_CI_: Mean centromeric index; CV_CI_: Coefficient of Variation of Centromeric Index; CV_CL_: Coefficient of Variation of Chromosome Length; m: metacentric chromosome; sm: submetacentric chromosome; st: subtelocentric chromosome; sat: satellite chromosomes; s.d.: standard deviation.

Morphometric parameters of chromosomes in eight taxa are also presented in Table 1. The karyotypes differed for the haploid chromosome length, the position of centromeres and satellite, and the karyotype asymmetry. Individual chromosome sizes varied from 0.89 to 4.08 μm. The shortest are observed in *S. koehneana* (0.89–1.79 µm) and *S. wilsoniana* (0.89–1.72 µm) while the longest is observed in *S. pohuashanensis* (2.05–4.08 µm). The total haploid length varies from 20.68 µm (*S. wilsoniana*) to 50.06 µm (*S. pohuashanensis*). Three taxa, *S. filipes*, *S. koehneana* and *S. wilsoniana*, have both very small and small chromosomes. Four taxa, *S. amabilis*, S. hupehensis var. paucijuga, *S. ovalis* and *S. scalaris*, have only small chromosomes. One taxon, *S. pohuashanensis* has both small and intermediate chromosomes.

**Figure 3.**
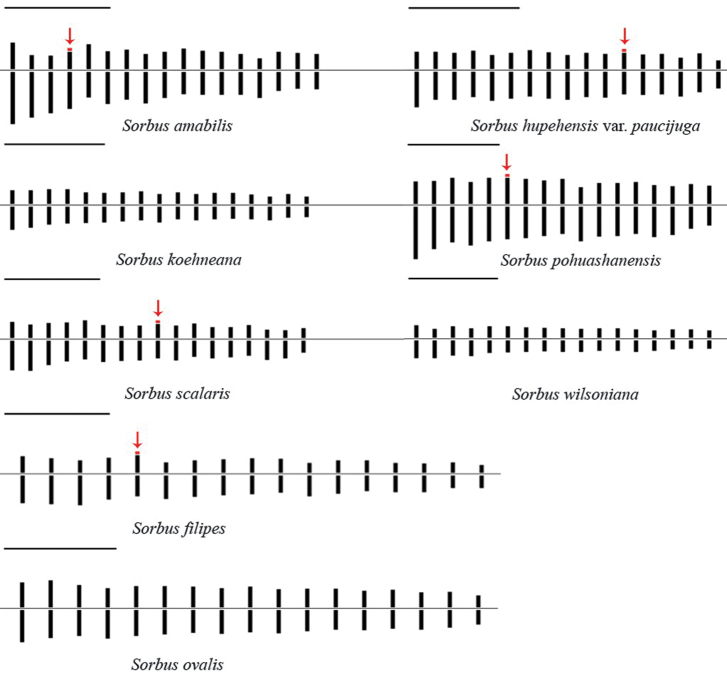
The mean haploid idiogram of the eight *Sorbus* taxa, based on median chromosome values. Arrows indicate secondary constrictions and satellites. Scale bars: 5 µm.

With respect to the position of the centromere, the chromosomes of the six taxa are metacentric or submetacentric. *S. amabilis* presents 9 metacentric (5, 8, 10–12, 14–17), 7 submetacentric (1, 3, 4, 6, 7, 9, 13) and 1 subtelocentric (2) chromosome pairs, and *S. ovalis* displays only metacentric chromosome pairs. A pair of satellites was observed in *S. amabilis*, *S. filipes*, S. hupehensis var. paucijuga, *S. pohuashanensis* and *S. scalaris*, with the satellites being located at the short arms of the fourth, fifth, twelfth, sixth and ninth chromosome pairs, respectively (Fig. 3).

According to the classification of Stebbins (Stebbins 1971), karyotypes of eight taxa are symmetrical and are classified as 1B (*S. koehneana* and *S. ovalis*), 2A (*S. wilsoniana*) or 2B (*S. amabilis*, *S. filipes*, S. hupehensis var. paucijuga, *S. pohuashanensis* and *S. scalaris*). CV_CI_ and CV_CL_ values of eight taxa ranged from 4.86 to 21.54 and 14.03 to 24.70, respectively (Table 1). CV_CI_ is a parameter indicative of the intrachromosomal symmetry. *S. ovalis* has the most symmetrical karyotype (CV_CI_ = 4.86), whereas *S. amabilis* has the least symmetrical karyotype (CV_CI_ = 21.54). CV_CL_ revealed that all taxa have little variations in chromosome size of the karyotypes. S. hupehensis var. paucijuga has the smallest CV_CL_ value (14.03) and *S. amabilis* presents the highest CV_CL_ value (24.70).

**Figure 4.**
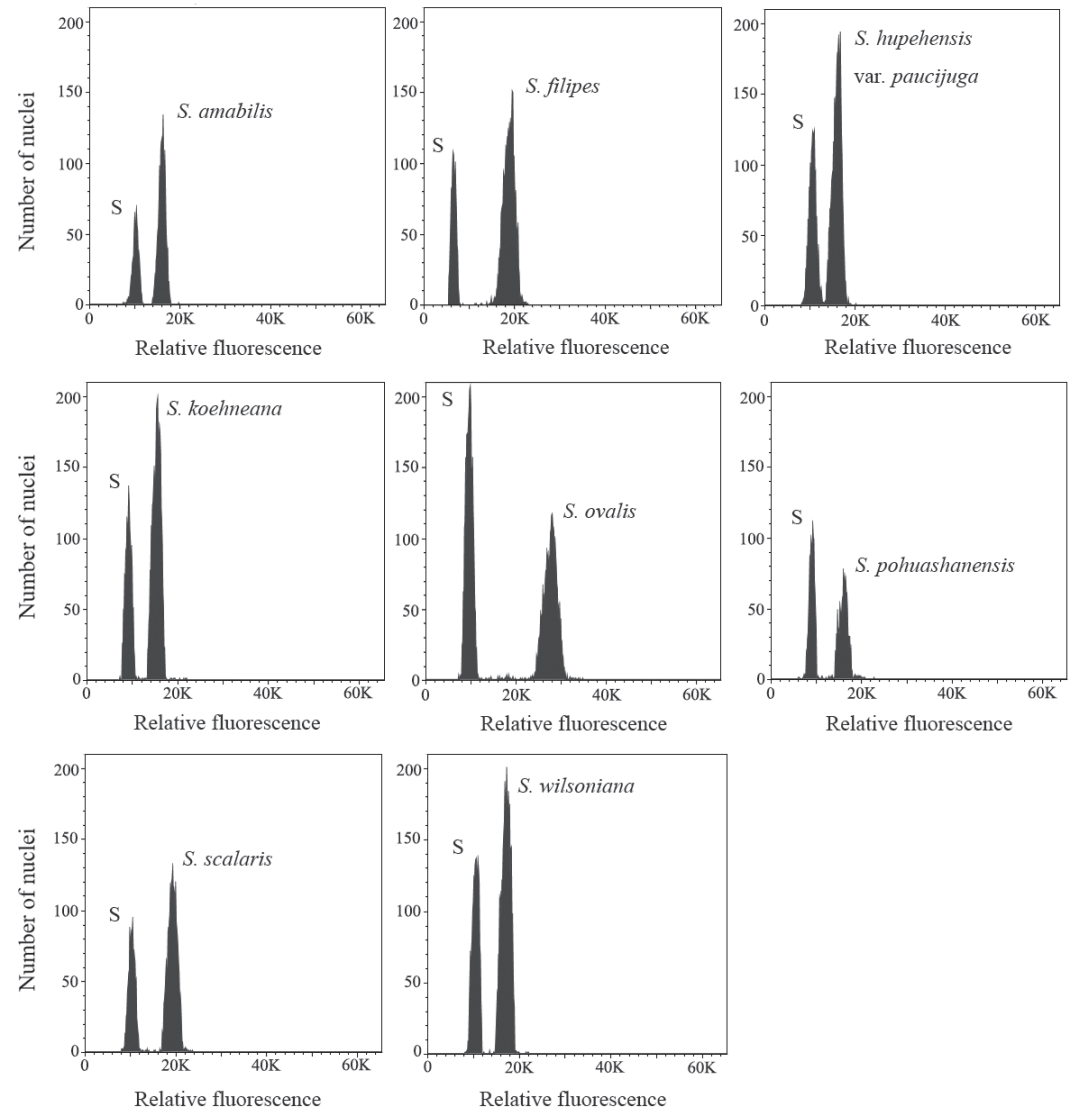
Flow cytometric histograms of each *Sorbus* species analyzed simultaneously with the internal standard Oryza sativa subsp. japonica ‘Nipponbare’ (S).

Genome size estimates of all the taxa from silica-dried leaves are shown in Table 1 and Figure. 4. The flow cytometric measurements of all taxa and the internal standards exhibit clear and sharp peaks. The coefficients of variation are lower than 5%, supporting the reliability of the flow cytometric assessments. The 2C-values range from 1.401 pg to 1.676 pg for diploid taxa. Two tetraploid taxa, *S. filipes* and *S. ovalis*, have 2C-values of 2.674 pg and 2.684 pg, respectively. 2C-values of tetraploids are approximately twice those of their diploid congeners and the relative DNA content correlate positively with the chromosome number (r = 0.982, P ≤ 0.0001). The 1Cx-values, which indicate the DNA content per genome, range from 0.700 pg to 0.838 pg in diploids and 0.669 pg to 0.671 pg in tetraploids. The correlation between values of 1Cx and chromosome number is negative (r = –0.687, P < 0.05).

Genome sizes of the eight taxa studied are reported for the first time. Our results are consistent with the chromosome counts and the variation reported for the genus in previous studies (Pellicer et al. 2012; Xi et al. 2020). Combining the results of previous findings with the results of this study, the total range of 2C-value for the genus for diploids and tetraploids is 1.401 pg ~1.631 pg and 2.674 pg ~ 3.226 pg, respectively. In addition, our data reflect that tetraploids (mean of 1Cx = 0.670 pg) have lesser values of monoploid genome size than diploids (mean of 1Cx = 0.773 pg), indicating a genome downsizing trend in the genus. The decrease in monoploid genomes after polyploidization is usually associated with the loss of repetitive DNA, such as retroelements or retrotransposons (Leitch and Bennett 2004; Bennetzen et al. 2005; Simonin and Roddy 2018).

In *Sorbus*, ploidy levels are closely related to the reproductive strategies: diploids are considered to propagate sexually while polyploids to propagate asexually (Jankun 1993; Aldasoro et al. 1998; Dickinson 2018). Although Lu and Spongberg (2003) recorded tetraploids *S. koehneana*, we have not found any polyploid specimen for the taxon in our sampling, so additional individuals of the taxon are required in future studies and the origin for tetraploids recorded should be considered. In Europe, modern taxonomic studies (Rich et al. 2010; R﻿obertson et al. 2010; Sennikov and Kurtto 2017) and descriptions of new species (Lepší et al. 2009; Vít et al. 2012; Néme﻿th et al. 2016; So﻿mlyay et al. 2017) are accompanied by counts of chromosome numbers or DNA ploidy levels, based on flow cytometry. New species also have been discovered constantly from China in recent years (Li and Gao 2015; Guo et al. 2016; Yin et al. 2017) and the difficulty in taxonomy of this genus will continue to increase. Thus, diversity in ploidy levels in Chinese native species needs further analysis of additional species and individuals.

## Conclusions

In this work, the first karyotype description and data about genome size are reported for eight *Sorbus* taxa. Consistent with previous studies, FCM has been found to be highly effective in estimating the relative DNA content of *Sorbus* species to infer ploidy. Further investigation on karyotype characteristics and ploidy levels of Chinese native *Sorbus* species is needed for a better understanding of the species’ relationships and origins.
